# Incorporation of Spatial Interactions in Location Networks to Identify Critical Geo-Referenced Routes for Assessing Disease Control Measures on a Large-Scale Campus

**DOI:** 10.3390/ijerph120404170

**Published:** 2015-04-14

**Authors:** Tzai-Hung Wen, Wei Chien Benny Chin

**Affiliations:** Department of Geography, National Taiwan University, No. 1, Sec. 4, Roosevelt Road, Taipei 10617, Taiwan; E-Mail: wcchin.88@gmail.com

**Keywords:** network analysis, community structure, spatial interaction, respiratory disease transmission

## Abstract

Respiratory diseases mainly spread through interpersonal contact. Class suspension is the most direct strategy to prevent the spread of disease through elementary or secondary schools by blocking the contact network. However, as university students usually attend courses in different buildings, the daily contact patterns on a university campus are complicated, and once disease clusters have occurred, suspending classes is far from an efficient strategy to control disease spread. The purpose of this study is to propose a methodological framework for generating campus location networks from a routine administration database, analyzing the community structure of the network, and identifying the critical links and nodes for blocking respiratory disease transmission. The data comes from the student enrollment records of a major comprehensive university in Taiwan. We combined the social network analysis and spatial interaction model to establish a geo-referenced community structure among the classroom buildings. We also identified the critical links among the communities that were acting as contact bridges and explored the changes in the location network after the sequential removal of the high-risk buildings. Instead of conducting a questionnaire survey, the study established a standard procedure for constructing a location network on a large-scale campus from a routine curriculum database. We also present how a location network structure at a campus could function to target the high-risk buildings as the bridges connecting communities for blocking disease transmission.

## 1. Introduction

Because respiratory diseases are mainly spread through interpersonal contact [1], class suspension, with the subsequent termination of interpersonal contact, is the most direct way to control severe respiratory diseases in a school setting. Class activities usually cause long duration face-to-face contacts, which is one of the important risk factors for the spread of respiratory diseases, such as influenza, in a school environment [2,3,4]. During the early stages of the novel influenza A/H1N1 pandemic in 2009, the Taiwan Centers of Disease Control (Taiwan CDC) suggested that if two students in the same class were diagnosed with confirmed H1N1 within a 3 day period, the school should suspend classes for 5 days [5]. Nevertheless, university students usually take courses in different buildings, and students’ classes are not restricted to a single building or classroom. Once severe respiratory disease clusters occur on campus, class suspension is not sufficient for disease control [6]. Understanding the structures of the student flow among classroom buildings at a university campus may be a vital clue to assessing the impact of disease transmission.

The concept of a social network is useful for understanding the complexity of interrelationships among people [7,8]. A social network is a graphic structure with social implications signified by nodes and links. Nodes can represent the people or organizations, whereas links can indicate the social relations between the nodes. Because social networks focus on analyzing the interrelationships among people or organizations, they can be used to investigate patterns of disease spread [9] and to provide effective ways of blocking disease transmission [10,11]. For example, Xu and Sui [8] analyzed how networks affected the spreading pattern of a contagion and the benefits of responsive vaccines. They also clarified the process of disease spread and its relation with social networks and simulated the variation of infection rates given different rates of vaccination, including a lack of vaccinations. Christakis and Fowler [12] investigated the source of infection within a friendship network on a university campus. They predicted the spread of disease using a random network and the friendship network and found that use of the friendship network could predict the spread of disease earlier than the random network.

**Figure 1:**
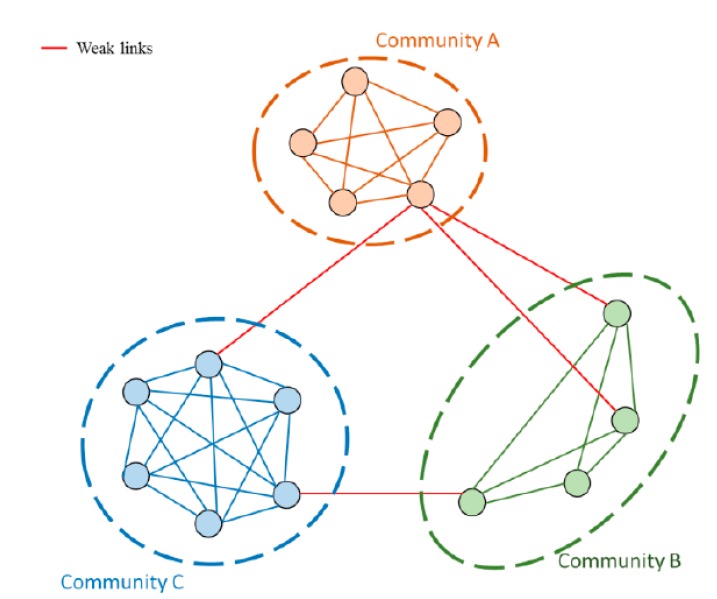
A group of nodes with tight connections constitutes a community whereas a weak link connects different communities. By identifying the connectivity of the links, the community structure can be depicted.

Furthermore, people usually gather together because of similar personalities, preferences and life circles, leading to the formation of communities (Figure 1). Social network analysis has also been used to analyze how people or organizations form clusters through behaviors as well as the impact of the network structure on people’s behaviors. Therefore, identifying communities within a network has been an important topic in the study of social sciences and epidemiology [13]. A group of people with tight connections constitutes a community, a network structure that accelerates the transmission rate. This is because within a community, people could reach each other by different paths, and thus interaction between the nodes within a community would be rapid and more intensive. Although some communities are more remote, pathogens can still pass from one community to another via nodes or links that act as bridges, which further propagate the distance and scope of the infection. Hence, understanding the structure of communities and using the concept as a control measure becomes the main procedure for disease control [14]. For example, Salath and Jones [14] used both actual networks and simulated networks to study how a community structure affected the proliferation of a contagion, finding that the outbreak always originated in well-established communities. Thus, restraining the infection area by blocking the links (critical links) or nodes (high-risk nodes) between communities should be an effective strategy to prevent the continuous spread of the pathogen into different communities.

The critical links, also called weak links, are the connections that act as bridges, connecting two independent communities [15] (Figure 1). The links bridging two communities indicated that pathogen could be carried from one community to another, and would magnify its contagious range dramatically. Consequently, identifying critical links is important not only because they are the most likely routes for pathogen transmission, but also because they are the medium for pathogens to spread to further locations [16,17]. Similarly, the nodes that are most often found between each pair of additional nodes would also be found between two communities [18], namely, high-risk nodes. Because high-risk nodes bridge two communities, removing these nodes by isolating or monitoring them would increase the separation between the communities [19]. However, the investigation and construction of the physical interpersonal contact network are difficult and are not only expensive in terms of data collection and sampling but also hard to maintain in terms of data quality. The lack of a complete interpersonal contact network has been an issue with the implementation of epidemic prevention and control measures.

Physical interpersonal contacts always occur in geo-referenced spaces [20]. Because spatial interactions describe the interrelationships among persons in geo-referenced spaces [21,22], a spatial interaction model, namely, gravity modeling, is a way of measuring the strength of interaction between geo-referenced spaces. In such a model, the interaction between spatial features would be proportional to their “attractions” and inversely proportional to the “impedance” between them. In a geographical analysis, the “attractions” and “impedance” are usually replaced by a quantity variable (population number, gross domestic product) and a distance variable (travelling path’s length, geometric distance) [23,24]. By combining spatial interactions and contact networks, a spatial interaction network could also function as a spatial pattern of geospatial contacts between people, which could be used to understand how a disease may spread in geo-referenced spaces. In other words, people who were sharing a living circle (where they were doing some activities) would increase the direct or indirect interactions, which would then influence the pattern of disease spreading [25]. Moreover, understanding the spatial diffusion patterns in a network of geospatial spaces would be useful for spatial targeting against epidemics. In other words, this model could provide insight into selecting high-priority nodes for removal.

Therefore, this study aims to understand the spatial structures among department buildings based on the students’ class-taking behaviors. A spatial interaction network model is constructed to represent the spatial intensity of student flow between buildings. Geo-referenced community structures of department buildings are identified as possible life circles at a university campus. By identifying these community structures among buildings, including the critical links and high-risk buildings, we were able to understand the role of critical links among communities and to use these findings to implement major prevention strategies targeting high-risk buildings as high priority of intervention for blocking pathogen transmission through on-campus contact.

## 2. Materials and Methods

### 2.1. Curriculum Database of Class Registration

Class activities cause long duration face-to-face contacts within indoor environment and could increase the risk of respiratory disease transmission [2,3,4]. Therefore, this study focused on analyzing spatial structures of class-taking behaviors. To represent the class-taking behavior, we used class enrollment data at a major comprehensive university in Taiwan during 2009. The data included several tables: a list of students and departments, a list of departments and buildings, course-classrooms (buildings) list, and student course enrollment records. Because we focused on the disease transmission within department buildings, outdoor courses were excluded from our dataset. Moreover, classes held on other campuses were also excluded. Thus, all indoor lecture/seminar courses that took place at the main campus were included in our dataset.

Our dataset covered 17,513 students, 3214 courses, 144,700 registration records, 6059 classes, the locations of 135 department buildings, and the relevant information for these departments. We summarized the data into several data points, including the total number of students who took classes in each building, the flow volume of students moving between each department building, and the geographical distance between each building.

### 2.2. Spatial Interaction Network Model

Students who attend courses in nearby buildings may share a similar life circle on the campus. In other words, students could have higher probability of contact with each other if the course-taking buildings are more nearby. To capture potential contact relationships between the departments, we set out to establish a standard procedure to construct a geo-referenced location network based on the students’ class-taking locations and the spatial interactions among department buildings. We used the spatial interaction model to measure the level of potential interaction between each pair of buildings. In this study, the “attractions” were the total number of students who took classes in each building, and the “impedance” was the squared distance between the two buildings (see Equation (1)):

(1) $$G\left(i,j\right)=\frac{M_{i}\;\times \;M_{j}}{d_{ij}^{2}},\;i\neq j$$

*G*(*i,j*) indicated the level of spatial interaction between building *i* and *j*. *M_i_* and *M_j_* were the total number of students enrolled in at least one class in building *i* and *j*, while *d_ij_* was the Euclideandistance between the two buildings.

The interaction between two buildings was restricted by the existence of students moving between the two buildings. For example, if no students from department A took a class in building B, the interaction should be zero. We investigated the number of students moving between each pair of department buildings, resetting the level of spatial interaction for pairs of buildings without any class activity to zero. Moreover, to capture the possible routes of disease transmission, stronger spatial interactions between the buildings could be regarded as having a higher possibility of involvement in pathogen circulation. Therefore, we reserved stronger spatial interactions in the first quarter as the links, while nodes represented department buildings in the spatial interaction network model.

### 2.3. Analysis of Community Structure

A community was formed by a group of department buildings with tight connections, which network structure would accelerate the transmission rate because the interaction between the nodes within a community would be more intensive because students could reach each other by several alternative paths. A critical link appears in the shortest path (the most simple and direct path connecting two nodes) that links each pair of nodes, implying that such a link can control the delivery of messages between nodes [16,17]. “Edge betweenness” [18] refers to the frequency with which each link appears in the shortest path between each pair of nodes (see Equation (2)). Because the shortest path is the fastest and the simplest route for diffusion, the link with the highest frequency of passing through by the shortest path would be the most “between” link for each pair of nodes. In network analysis, these high edge betweenness links are also known as weak links:

(2) $${\text{E}}_{b}\left(AB\right)=\frac{\delta_{i-AB-j}}{\delta_{i-j}},\;i\neq j$$

where $E_{b}\left(AB\right)$ is the edge betweenness of the link between node *A* and *B*, $\delta_{i-AB-j}$ is the frequency that *link*−*AB* appears in the shortest path between each pair of nodes *i* and *j*, and $\delta_{i-j}$ is the total number of the pair of nodes.

Links with greater edge betweenness play more important role as “bridges”. If all the bridges were removed, the network would be separated into several “islands”, (smaller pieces of networks). In social network analysis, these “islands” are called communities. Communities are composed of tightly connected nodes that have only a few links to other communities. Our study adopted the GN algorithm which was developed in 2002 for identifying communities. This algorithm repeats the process of removing the link (or links) with the current highest edge betweenness to progressively reveal the underlying community structure [26]. When a link with a high edge betweenness was removed, the network could be separated into two sub-networks, which indicated that two communities were found in the current generation. The communities would then be separated into smaller sub-networks in the next generation.

### 2.4. Assessment of Intervention Strategies

The study developed two network-based epidemic control strategies to assess the impact of location network structure on disease transmission, including blocking geo-referenced critical links between communities and the removal of high-risk buildings from the network. Blocking the critical links and removing bridge nodes would break the connection among communities. It could increase the degree of separation of the location network and decrease interactions among department buildings.

**Figure 2:**
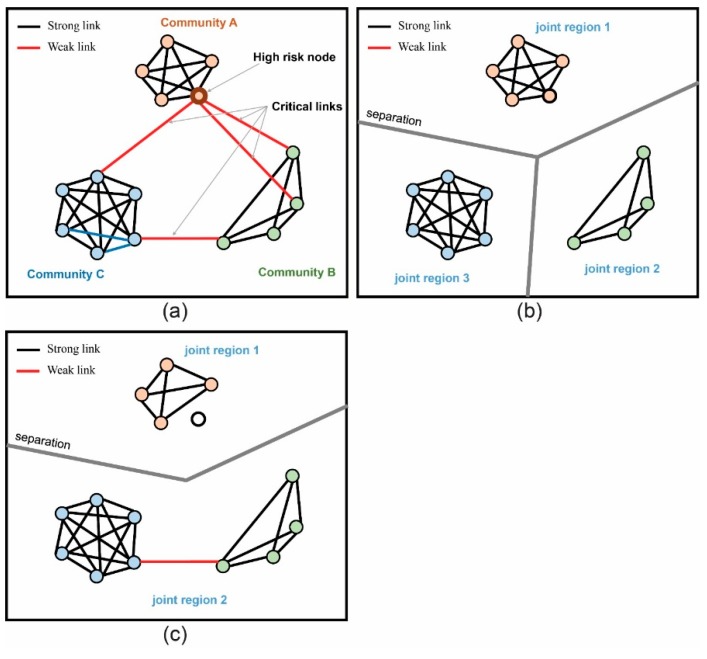
the illustration of join-region control for blocking disease diffusion: (**a**) Identifying communities, critical links and high risk node; (**b**) By removing all critical links, the communities were separated and formed three independent regions; (**c**) By removing high risk node, the communities were also separated and formed two regions. Since regions were isolated with each other, the disease would not diffuse from one region to another.

#### 2.4.1. Blocking Geo-Referenced Critical Links

After the communities were identified, the links connecting two nodes from different communities were identified as critical links (Figure 2a). Our study adopted the concept of joint-region control by cutting off the critical links to minimize the risk of rapid disease spread. Blocking critical links could effectively inhibit the disease range (Figure 2b).

#### 2.4.2. Removal of High-Risk Buildings

We also defined the high-risk buildings as the nodes (buildings) with a high node betweenness centrality [18]. Like edge betweenness, node betweenness refers to the frequency that a node appeared on the shortest paths between each pair of other nodes (Equation (3)). Thus, in the contact network, the building with the highest betweenness would be the most “between” building, which acts as a bridgebetween other buildings:

(3) $$N_{b}(k)=\frac{\delta_{i-k-j}}{\delta_{i-j}},\;i\neq j\neq k$$

where $N_{b}(k)$ is the node betweenness of the node *k*, $\delta_{i-k-j}$ is the frequency that node *k* appears in the shortest path between each pair of nodes *i* and *j*, and $\delta_{i-j}$ is the total number of the pair of nodes.

Removal of the high-risk buildings aimed to increase the degree of separation by isolating or monitoring high-risk buildings from the contact network (Figure 2c). The degree of separation is the average number of links in the shortest path between each pair of nodes (Equation (4)):

(4) $$D_{s}=\frac{\sum \sum N_{link}(i,j)}{2\times \delta_{i-j}},\;i\neq j$$

where $D_{S}$ is the degree of separation, $N_{link}(i,j)$ is the total number of links in the shortest path that connect node *i* and node *j*, and $\delta_{i-j}$ is the total number of pair of nodes. First, we calculated the betweenness of each building and ranked the buildings by their betweenness, from highest to lowest. Then, starting from the top-ranked building, we removed each building one by one and calculated the degree of separation of the remaining network and its variation from the previous network. In order to assess the strategies of removing nodes for blocking transmission, the random removal of nodes were tested with Monte Carlo simulations to evaluate the changes in the degree of separation. The envelope of the random removal was established by Monte Carlo simulations in comparison with the strategy of removing high-risk nodes.

## 3. Results

### 3.1. Community Structure

**Figure 3:**
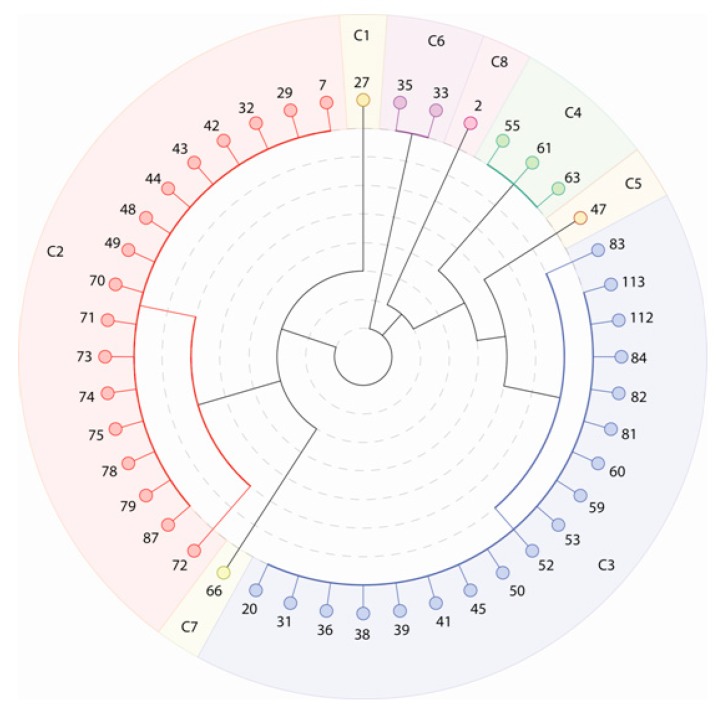
The communities of the location network at the campus.

By reserving the stronger spatial interactions of the first quarter as the links of the network, some of the department buildings with minor interactions became isolated. There were 68 department buildings remaining in the location network, with eight identified communities (Figure 3). As shown in Figure 3, most of the nodes were grouped into two major communities (C2 and C3); the other six communities contained only one to three nodes.

**Figure 4:**
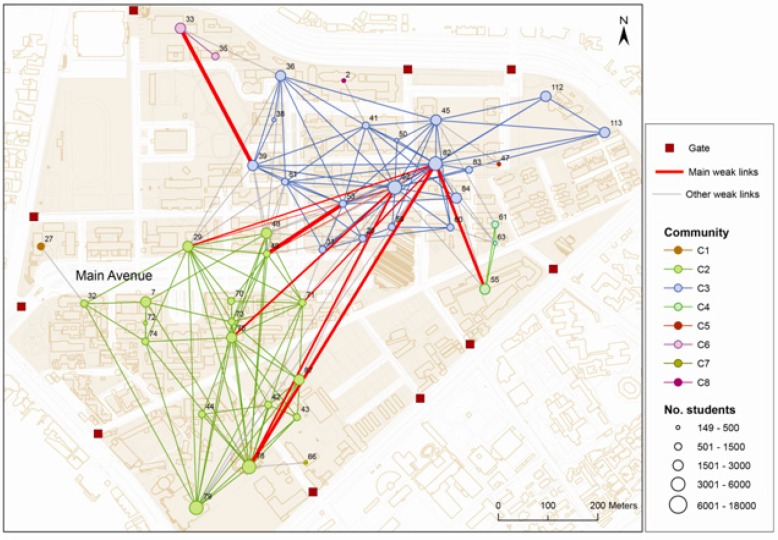
Mapping the geo-referenced communities and the circle links from the location network. The red links indicated the major critical links. The widths of the red links are proportional to their edge betweenness.

Figure 4 shows the spatial distribution of the eight communities. Because the network model was constructed based on the spatial interactions between buildings, the geographic locations of all nodes in each community were spatially clustered together.

In general, the two major communities were separated by the main avenue: the southern community (C2) and the northern community (C3). The other six communities were located at the margins of the campus, each adjacent to different side entrances to campus. As shown in Figure 4, the link density was higher within each community. The link density represented the intensity of interactions between a building and the other buildings, while the total number of students in the building represented the extent of class activity within the building. The buildings within the same community had stronger and more intensive interactions with each other, implying that the students who attended a class in a community might be influenced (or be infected) by other students who also attended a class in the same community. Therefore, if a building was proven to be infected, the other buildings in the same community should be treated as potentially exposed. On the other hand, our results also indicated that the link density was positively correlated with the total number of students in each building, implying that the places with higher intensity of interaction would also be the places with more students. Thus, these places might be at a higher risk of transmission.

### 3.2. Network-Based Epidemic Control Strategies

The critical links (weak links) in this study indicated that some students chose to attend classes in buildings in different communities, and both of the buildings (the origin building and the destination building) had a higher total number of students. Figure 4 shows the critical links between the buildings in different communities. The red links in the figure are the critical links between the two major communities. Some of these critical links connected the same pair of communities and also connected different sub-community clusters in the southern community to the northern community. Furthermore, our results also showed that for some of the critical links, the locations of some buildings that were connected with high-betweenness edges were close. This might be because the location network was built with the consideration of the distance decay effect, which was embedded in the spatial interaction model. This implied that although some of the nodes (buildings) located at the edge of the two communities were close to each other, the total number of students for most buildings was not high enough to form a relatively high spatial interaction. Therefore, those links with a high spatial interaction became the mandatory path connecting the two communities. Figure 4 also demonstrates that those critical links between the farther south sub-community cluster and the northern community have lower edge betweenness. This might because some alternative route existed within the southern community, which lowered their edge betweenness. Identifying critical links could assist in slowing the transmission among communities to reach joint-region prevention. Inhibiting these core links could lower the risk of transmission from boundary to central communities.

**Figure 5:**
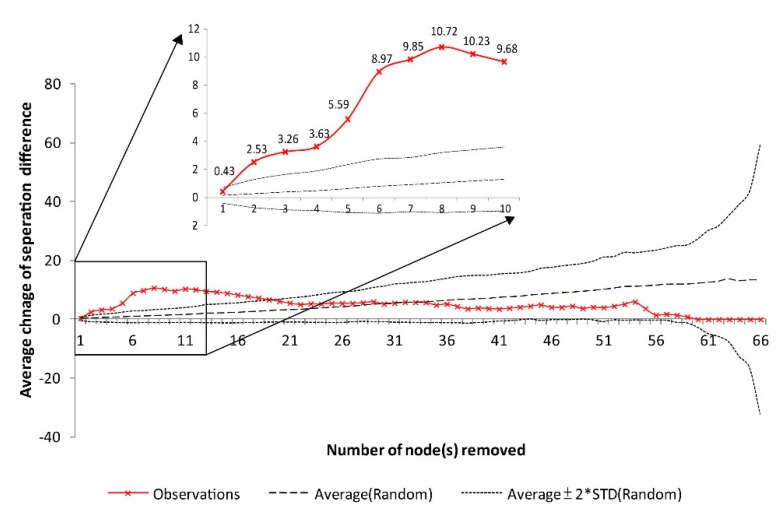
The red line is the changes of the degree of separation of the remaining network while each node with the currently highest node betweenness was removed. The dash line is the results of random removal.

Figure 5 shows the changes in the degree of separation each time a node was removed. Our results indicate that the changes in the degree of separation become more notable following removal of the building with the second highest betweenness; the difference following random removal reached as high as 10.72 after the first eight buildings were removed, indicating the maximum marginal effect of the degree of separation revealed with the elimination of the top eight buildings with the greatest node betweenness. After the 8th building was eliminated, the increment of the separation degree slowed, with some fluctuation. In other words, after the 8th building was removed, further removal did not greatly increase the degree of separation of the network’s topological structure. The changes in the degree of separation by removing the top 20 buildings with the highest betweenness were all significantly higher than the change observed with random removal. These results showed that the impact of removing buildings with high betweenness to the degree of separation was more significant than random removal (Figure 5) and that the first eight buildings were the most important for increasing the degree of separation. Therefore, spatial targeting at these buildings may be first high priority in response to disease outbreak. The imapcts of epidemics could be alleviated through monitoring and blocking entry to these buildings.

## 4. Discussion

University students construct complicated contact relationships on campus by taking classes in different department buildings. Once a respiratory disease outbreak occurs, some control measures, such as class suspension, are not sufficient to stop the spread of an infectious disease. Our study proposes a network-based epidemic control strategy with the design of a location network and spatial interaction models. Analysis of location networks takes the perspective of a topology structure to distinguish the spatial patterns of the network and to implement control measures when the sources of infection are unknown. Furthermore, preventions based on the community structures built in this study could capture the principle of neighboring life circles, which means that students of the same life circle have higher rates of contagion due to closer connections. This concept could reflect the nature of the spreading patterns of respiratory disease. We will further discuss some issues of the aspects of constructing contact networks, identifying community structures and implications to epidemic control strategies.

Collecting social contact data involves issues regarding the data sources and sampling. Therefore, rather than using a questionnaire survey, student registration records from a curriculum database was used to construct the location network, not only because course-taking is one of the most typical activities at universities but also because class enrollment records are routine for each student, which means the contact relationships could be derived without additional cost and effort. In addition, by using the student’s class enrollment records, we collected the student population characteristics without using student IDs, thus maintaining students’ anonymity in our study. Using the student’s class enrollment records precluded the potential problems of data sampling or privacy issues.

Methodologically, contact relationships are always embedded in geo-spaces. However, most network studies have concentrated on topological structures of the network and ignored the properties of geo-referenced locations in the network model [8,10,11]. Previous studies analyzing community structures also have focused on clusters of individual social relationships, producing networks clustered around actual geographic locations to assess the impact of spatial distributions to social relations [27,28]. Moreover, the intensity of interactions among locations could also a vital clue to understanding disease transmission [25]. The distance between individuals at campus would influence contact frequency and the probability of disease transmission from one to another [13,29]. Therefore, we proposed the framework of incorporating spatial interactions in location network structures. Spatial interactions can reveal the extent of class activity in each building and the flow volume and impedance of students moving between buildings. The spatial interactions indicated the level of sharing life circle among locations, which could reveal the extent of class activity in each building and the flow volume and impedance of students moving among buildings. Our study used the distance between buildings as the impedance of contact frequency. Students may share the similar life circles if classroom buildings are close to each other, and it would be easier to be infected by other infective peoples in the buildings within the same life circle [29]. Once a respiratory disease clusters occur, the extent of class activity could contribute to the scale of the pathogen transmission. Therefore, the spatial interaction network model proposed in this study could capture the geographic ranges of respiratory disease diffusion from the infection source’s surrounding environment [1,30]. Communities within the campus identified in this study also revealed the characteristics of geographic concepts of life circles (proximity), distance decay effects and transmission of respiratory disease [31,32].

The major campus of the comprehensive university used in this study covers an area of 1.1 km^2^ and includes 10 colleges and 44 departments. The department buildings are widely spread over the campus. Students have a lot of flexibility in terms of class choice, which means they can select courses based on their own interests and preferences. Based on these characteristics, the contact structures within the campus are complicated in terms of the spatial scale, heterogeneities of curriculum and students, similar to many other comprehensive universities around the world. This study established the standard procedure for establishing a campus geo-referenced location network, including data collection, derivation of contact relationships from a curriculum database, construction of a network model and geographic analysis and visualization of communities and critical links, which can be applied to other similar universities and colleges around the world to implement strategies of spatial targeting against the transmission of severe respiratory diseases.

In this study, one of the proposed network-based control measures was blocking critical links between communities. From the perspective of network topology, we should remove the critical links from the network until there is no connection between communities [15]. Blocking critical links refers to controlling the volumes of floating individuals along the connection and to comprehensively monitor the class behaviors in the connected building, such as monitoring students for fever, promoting the use of protective masks and examining the physical conditions of students [33]. Therefore, blocking critical links could be effective in blocking the spread of disease between communities.

However, another key question in disease control is the issue of how to stop the disease spread within a community at the initial stage of an epidemic. To block disease spread by increasing network separation, nodes acting as “bridges” with high betweenness should be blocked. Targeting the buildings with high betweenness instead of with high connections is more effective [14,34]. In this study, we removed the high-betweenness buildings to increase the level of separation as another network-based control measure. The most useful measure on campus is to block entry to all classroom buildings, that is, to enforce class suspension [6]. However, this strategy is often costly and thus its effectiveness on university campuses is limited due to limited resources. Therefore, by experimenting with the continuous removal of nodes in this study, we measured the marginal variations of network separation each time a single node was eliminated. We found that removing the first eight nodes with the highest-betweenness from the network can significantly damage the network structure, findings which are concordant with previous studies by Watts and Strogatz [19] and Centola and Macy [35]. In this study, we found that most of the high-betweenness nodes were also bridges connecting two major communities on campus. The links and nodes with high-betweenness can be regarded as critical locations as bridges connecting communites. In conclusion, our findings imply that identifying the life circle where the source of infection is located on campus can effectively shrink the control areas and improve the efficiency of intervention and resource dispatch. Spatial targeting of high-betweenness department buildings within the community should be the first priority for intervention once severe respiratory disease clusters are reported.

There are still some limitations to this study. First, temporal fluctuations of class activities are not incorporated in the study design. Different semester year may have different course-taking patterns. Temporal fluctuations of spatial interactions among department buildings could provide more information on dynamics of the location network. Second, the data used in this study focused on class activities because of the characteristics of disease transmission, including indoor environment, long-duration of contacts, close-proximity among individuals. Other locations or buildings such as dining halls, dormitories, and libraries could also be potential destinations. However, those movements are not daily routines for college students as the class-taking schedule and the flow data of students (to or from) these locations would be difficult to be collected. The flow among these locations would not incorporate in the study design and it could cause missing important bridge nodes at campus. The comprehensive mobility data warrant further investigation. Third, the study covered only the structure of students’ flows, which implied that other kinds of people, such as faculty members and staffs, were not incorporated. Our study captured the class activities of the students, which are the majority on a university campus, however, the mobility patterns of faculty members could be another critical link among locations because they could teach courses at different classroom buildings at one day. These data is worth collecting in further studies for constructing a more comprehensive location network.

## 5. Conclusions

Understanding the spatial interactions among locations is one of important clues to blocking respiratory disease transmission on campus. In this study, by constructing the location network, we captured the concept of spatial interactions and analyzed the structures of the network. Rather than conducting a questionnaire survey, this study used a routine curriculum database to collect the class activities, derive spatial relationships among classroom buildings, construct a network model and identify of geo-referenced communities, critical links and risk buildings. Location networks analysis takes the perspective of a topology structure to distinguish the spatial patterns of the network for implementing control measures when the sources of infection are unknown. Furthermore, preventions based on the community structures could capture the concepts of neighboring life circles, which means that students of the same life circle have higher rates of contact due to their closer connections. The high-risk locations such as the bridges connecting communities could be spatially targeted as first priory of intervention in response to a disease outbreak. We believe the framework is also appropriate to be applied to other similar universities and colleges around the world to implement strategies of spatial targeting against the transmission of severe respiratory diseases on campus.
